# Evaluation of Electromagnetic Interference and Exposure Assessment from s-Health Solutions Based on Wi-Fi Devices

**DOI:** 10.1155/2015/784362

**Published:** 2015-01-06

**Authors:** Silvia de Miguel-Bilbao, Erik Aguirre, Peio Lopez Iturri, Leire Azpilicueta, José Roldán, Francisco Falcone, Victoria Ramos

**Affiliations:** ^1^Telemedicine and eHealth Research Unit, Health Institute Carlos III, 28029 Madrid, Spain; ^2^Electrical and Electronic Engineering Department, Universidad Pública de Navarra, Pamplona, 31006 Navarra, Spain

## Abstract

In the last decade the number of wireless devices operating at the frequency band of 2.4 GHz has increased in several settings, such as healthcare, occupational, and household. In this work, the emissions from Wi-Fi transceivers applicable to context aware scenarios are analyzed in terms of potential interference and assessment on exposure guideline compliance. Near field measurement results as well as deterministic simulation results on realistic indoor environments are presented, providing insight on the interaction between the Wi-Fi transceiver and implantable/body area network devices as well as other transceivers operating within an indoor environment, exhibiting topological and morphological complexity. By following approaches (near field estimation/deterministic estimation), colocated body situations as well as large indoor emissions can be determined. The results show in general compliance with exposure levels and the impact of overall network deployment, which can be optimized in order to reduce overall interference levels while maximizing system performance.

## 1. Introduction

Mobile communication devices have become omnipresent in almost all fields of daily life, including home, health, and labor environments and even the displacements. Since the point of view of the healthcare all these fields of application are not disjoint and are involved in the concept of smart health (s-health). The widespread use of wireless technologies and the great interest in ubiquitous communications, seeking connection anywhere and anytime, have meant the emergence of the concept of s-Health as the results of the natural synergy between mobile health (m-health) and smart cities, from the information and communications technology (ICT) perspective and also from that of individuals and society [1].

In healthcare environment, the introduction of wireless communication systems has promoted the improvement in the efficiency of patient care and health management. One of the scenarios of applicability of wireless communication systems in healthcare environments are the ubiquitous healthcare networks [2] that allow the patient care and the assistance regardless their location.

Body area networks (BAN) are used in several healthcare scenarios: ambulances, emergency rooms, operating rooms, postoperative recovery, clinics, and even homes. BANs are characterized by the following components: the network nodes are sensors (or telemetry devices) that measure biological parameters and the router that collects information detected by sensors and then transmit it to the control center. Regarding the type of interfaces that form the BAN, the following types are considered: short range interface that connects the telemetry devices (or sensors) with the router or gateway, and wide area networks (WAN) that allow connectivity between the router and the control center. Ideally, seeking greater ubiquity in patient care the network interfaces are wireless.

Typically BAN devices are incorporating Wi-Fi interfaces to communicate with the router and contain low-powered radiofrequency (RF) transceivers that support wireless local area networks (WLANs). The component of the BAN that acts as a router is provided with a Wi-Fi interface and a WAN interface to route the patient information to the control center. In this context the Wi-Fi devices generally work in proximity to persons, which can lead to an excessive perception of risks related to electromagnetic field (EMF) exposure. Moreover, interference with other devices within this framework which can lead to potential malfunction requires assessment.

This paper deals with the evaluation of the electric field strength levels from Wi-Fi systems, both in situations in which the transceivers are colocated with the body and within a larger indoor environment. A near field measurement procedure is followed in the initial case, whereas a deterministic simulation approach, employing 3D ray launching is employed in the latter case, owing to computational complexity constraints. The emission levels have been obtained in far field and near field conditions in order to test the compliance with the recommended standard to assure the safety of people. The European standards are essentially based on the guidelines formulated by the International Commission on Nonionizing Radiation Protection (ICNIRP), a nongovernmental organization, formally recognized by the World Health Organization (WHO), which establish exposure limits by taking into account ascertained health effects.

ICNIRP defines limits that have been established in the great majority of countries in the world. Two classes of guidance are presented [3]: basic restrictions of ICNIRP are derived from the considerations related to established adverse health effects and are given in terms of dosimetric quantities, that is, induced current density for low frequency and specific absorption rate (SAR) for radio frequency and microwaves. As these quantities cannot be measured outside the body, reference levels are provided for practical exposure assessment to determine whether the basic restrictions are likely to be exceeded. Reference levels are given in terms of radiometric quantities, such as electric and magnetic field strengths. Compliance with the reference levels will ensure compliance with the relevant basic restriction [4]. If the measured or calculated value exceeds the reference level, it does not necessarily imply that the basic restriction will be exceeded. However, whenever a reference level is exceeded it is compulsory to test compliance with the relevant basic restriction and, hence, to determine whether additional protective measures are necessary.

Compliance with the present guidelines may not implicitly avoid interferences with, or effects on, medical devices such as metallic prostheses, cardiac pacemakers and defibrillators, and cochlear implants [5]. It is worth noting that interference with pacemakers may occur at levels below the recommended reference levels.

In this context, it is worth noting the new Directive 2013/35/EU of the European Parliament and of the Council of 26 June 2013 on the minimum health and safety requirements regarding the exposure of workers to the risks arising from physical agents. The limit values for exposure to electromagnetic fields and the levels at which the employer must take action must now be based on the new, more stringent recommendations of ICNIRP [6].

Within the framework of s-Health, ambient assisted living and other context aware scenarios, the impact of the potentially massive use of wireless transceivers, mainly in Industrial, Scientific and Medical bands must be correctly assessed, in terms of electromagnetic exposure as well as interference emission compliance. The elaboration of new directives such as 2013/35/EU require in depth analysis on the use of such devices.

The paper is structured as follows: Section 2 will describe the near field measurement setup and results; Section 3 is devoted to indoor characterization, simulation and measurement; Section 4 provides discussion on the results obtained, finally leading to the conclusions.

## 2. Near Field Characterization

**Figure 1:**
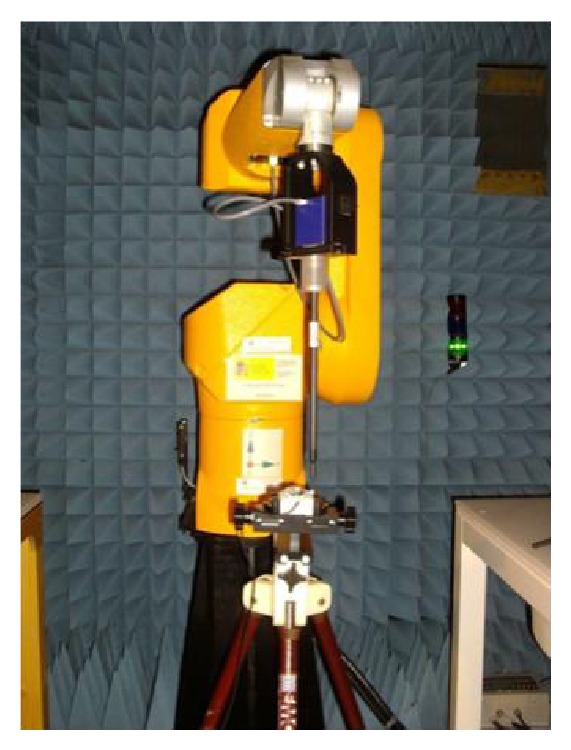
Near field measurement setup based on a DASY5PRO near field porbe and a tripod holding the device under test.

The first step in the characterization of potential radiated interference from WLAN transceiver elements within a context aware scenario is to analyze near field behavior. Thus, potential impact to implantable devices as well as impact on other elements which form part of a body area network can be determined. Initial measurements were performed with the aid of a DASY5PRO automated system with a coupled E-field probe, within an anechoic chamber, as shown in Figure 1 [7].

**Figure 2:**
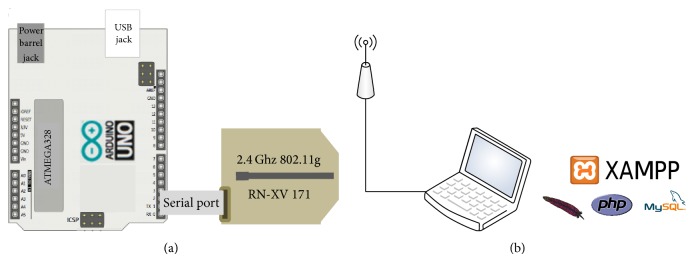
Components of the system to generate the communication from the Wi-Fi module. It consists in a Wi-Fi module connected to an Arduino microcontroller and an access point connected to a Xampp application server.

The specific Wi-Fi module was a WiFly GSX 802.11 b/g wireless LAN module that operates with the protocol 802.11 g, whose maximum allowed power is 10 dBm. A specific architecture to generate traffic from the Wi-Fi module was implemented in order to operate the employed Wi-Fi module, depicted in Figure 2. Transmission routines of the Wi-Fi module have been programmed with Arduino, and a specific connection is established with an auxiliary access point (AP).

The AP is connected to a laptop where specific software controls the traffic received from, and transmitted to, the Wi-Fi module. The AP and the laptop are connected through an Ethernet connection. The control functions of the communication between the module and the AP have been implemented through a Xampp Server, installed in a laptop. The Xampp Server is an independent server platform, which consists on a MySQL database, an Apache web server, and interpreters for scripting languages such as PHP. The server allows setting the operating parameters of the AP, receives the data sent by the module, and establishes the connection with the database to store the received data.

The E-field measurements were taken in the interpolated points belonging to a predefined grid whose dimensions are 8.1 cm × 8.1 cm × 4.1 cm, with differential distances of *dx* = 1 mm, *dy* = 1 mm, and *dz* = 1 mm. The E-field levels were measured in points of the predefined grid that belong to the three planes: (*x*, *y*), (*x*, *z*), (*y*, *z*).

At 2.4 GHz, the wavelength is about 12.5 cm, which means the reactive near field extends to around 2 cm from the source. Taking into account that the length of the antenna of the Wi-Fi module is 3 cm, the radiating near field extends no further than around 1.44 cm at 2.4 GHz. The minimum distance between the probe and the antenna is 2 mm, so the great majority of the measurements during this work were made in the far field region with respect to the source.

**Figure 3:**
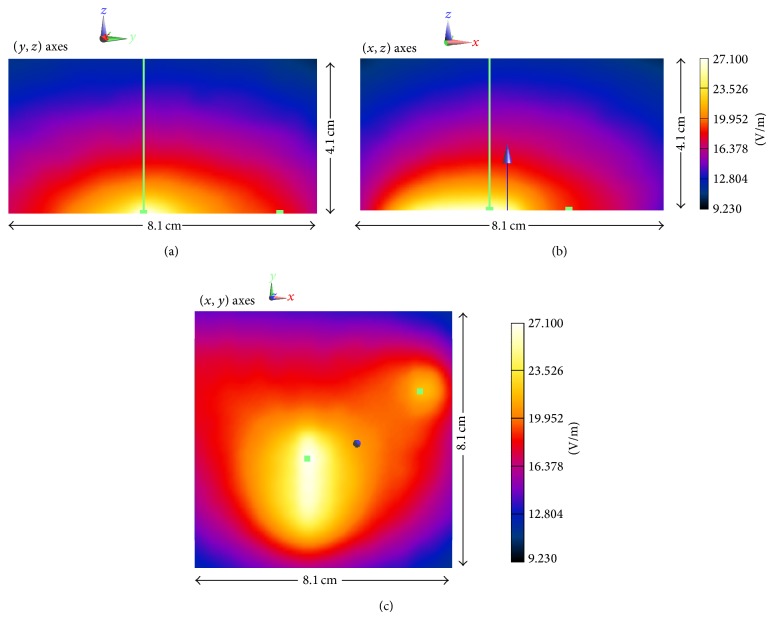
E-field around the tested device in the (*x*, *z*), (*y*, *z*), and (*x*, *y*) planes.

The obtained E-field values were compared with the thresholds of the recommended exposure levels (ICNIRP-98) [3] and the thresholds for the safety and basic performance of the electromedical equipment (IEC 60601-1-2) [8].  Figure 3 shows the E-field values in the three axes: (*x*, *z*), (*y*, *z*), and (*x*, *y*).

As it can be seen from the results obtained from the near field measurement setup, the highest measured level of the E-field is 27.1 V/m, which exceeded the most restrictive value of 3 V/m that is established in the International Electrotechnical Commission Standard of Electromedical Devices [8]. Therefore, use of the proposed transceiver must be carefully evaluated in terms of maximum allowed transmit power, in order to comply with previously stated guidelines. These near field results will be complemented with emission and interference estimation in a conventional indoor scenario in the following section.

## 3. Deterministic Indoor Characterization of Wi-Fi Transceiver Emission

The quantification of the E-field in the proximity of the device is enough for the assessment of EMI or exposure level analysis for many study cases, as the radiated E-field level is required for a complete scenario. These scenarios are usually complex indoor scenarios, where the radio wave propagation is affected by electromagnetic phenomena such as reflection, refraction, diffraction, and different effects due to multipath propagation. In order to pursue this issue, an in-house developed 3D ray launching algorithm, based on geometrical optics (GO) and geometrical theory of diffraction (GTD), has been used. This method is a midpoint between the analytical methods, which require low computational cost and provide limited accuracy [9, 10], and full wave techniques such as FDTD (finite-difference time-domain) or MoM (method of moments), which exhibit precise results but require high computational cost [11], which in many instances renders the approach unfeasible. Therefore, the presented deterministic 3D ray launching method, implemented in-house at the Public University of Navarre, offers an adequate compromise between calculation time and accuracy, and it has been previously validated in several complex indoor scenarios, for different applications such as the analysis of wireless propagation in indoor scenarios [12, 13], EMI analysis [14], or electromagnetic dosimetry evaluation [15].

In order to validate the presented in-house ray-launching software within the indoor scenario where the WiFly module will be tested, simulations have been made and radiopropagation measurements have been taken. The chosen scenario is a typical office-laboratory environment that can be found at the research building of the Public University of Navarre.

**Figure 4:**
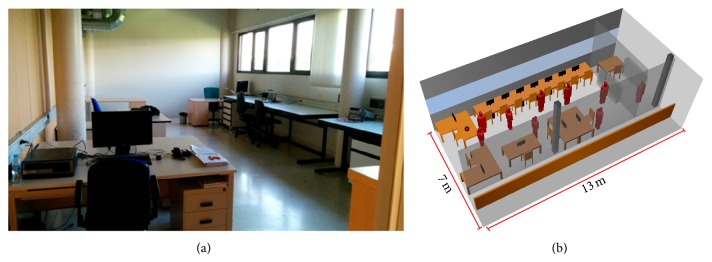
Laboratory of the University Public of Navarre (a) and its 3D representation (b) where the situation of WiFly is depicted (red point) and people have been randomly introduced.

Figure 4 shows the real scenario and its schematic representation considered for simulations. The scenario has the inherent complexity of this kind of indoor environments, with different types of walls (concrete and plywood), chairs, computers, tables, and so forth. The real dimensions of the objects within the scenario as well as their material properties (dielectric constant and loss tangent) at the frequency range of operation are considered by the 3D ray launching algorithm. Other parameters that have been set for the simulations are summarized in Table 1.

Note that the schematic image of the scenario in Figure 4(b) is populated with people. However, the simplified human body model is only used in the simulations where the real device is considered and, therefore, the following results belong to the empty room.

**Figure 5:**
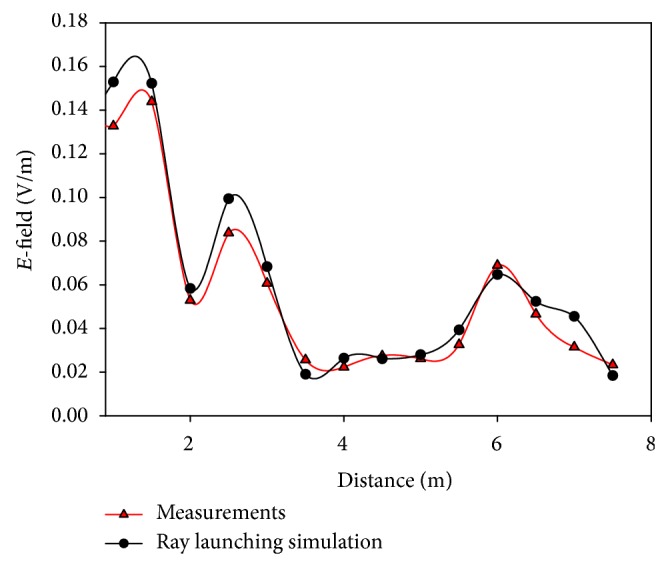
Received electric field values versus distance of measurements and ray launching simulation for the first control experiment.

**Figure 6:**
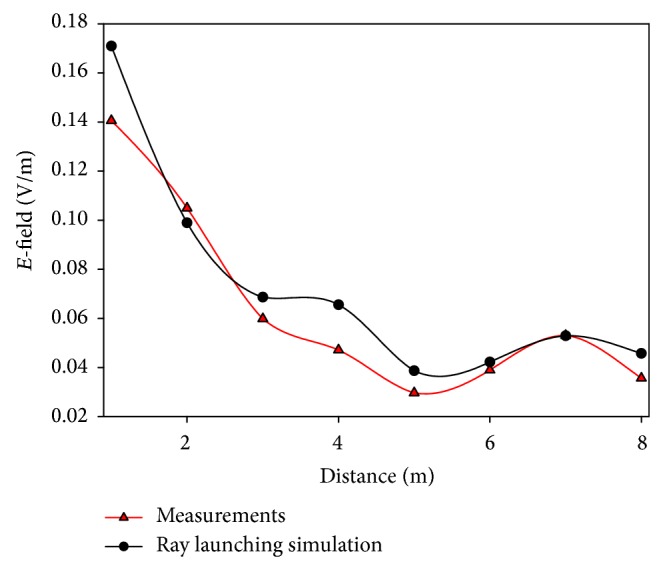
Received electric field values versus distance of measurements and ray launching simulation for the second control experiment.

As the WiFly devices operate at ISM 2.4 GHz band, two different simulations have been launched for two different frequencies within this range: 2.4 GHz and 2.45 GHz. The transmitter has been placed randomly on a table (at coordinates *X* = 9.94 m, *Y* = 4.5 m, at 0.60 m high) and the transmitted power has been set to 10 dBm, the maximum value allowed by the motes. In Figures 5 and 6 the comparison between the simulation results and measurements for randomly chosen linear paths within the scenario is shown, for 2.4 GHz and 2.45 GHz, respectively. Good agreement between measurements and estimated values can be seen, with a mean error of 0.0027 V/m at 2.4 GHz and 0.0092 V/m at 2.45 GHz.

The measurements taken for the comparison shown in Figures 5 and 6 have been carried out with a RF signal generator with a monopole antenna, emulating a transmitting device, and a N9912 Field Fox portable spectrum analyzer of Agilent, which provided the peak value for each measurement point.

Once the correlation between estimated values and measurements has validated the simulation method within the presented scenario, the study with the WiFly device has been carried out. The red point in Figure 4(b) indicates the location where the device has been placed, emitting with a power of 10 dBm. The received power has been measured placing the Fieldfox RF Analyzer every one meter in front of the device.

**Figure 7:**
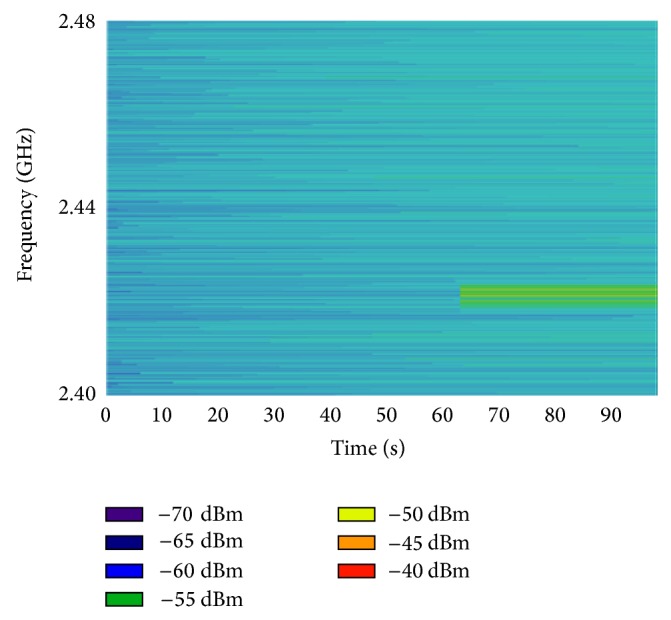
Measured spectrogram when the WiFly transceiver is disconnected (background power spectrum of the scenario).

The device usually needs to communicate with a data base as it can be shown in Figure 3; however, the power contributed by the access point is larger than the signal emitted by the wearable device. With the aim of accounting only for the WiFly transceiver, the AP has been switched off and the power received when the device is signaling. Nevertheless, the power emitted by other wireless networks must be considered. With this aim the spectrogram of the power received in the whole Wi-Fi band has been measured and it is depicted in Figure 7.

It is visible that in one of the first channels where Wi-Fi works a device is transmitting. Considering that the Wi-Fi module is programmed for working in the channel 11 (2.462 GHz), this problem is avoided and consequently the measurements can be carried out without interferences.

Since the exposure level and SAR values are directly related with the human body, not only has the empty scenario been considered in simulation, but also people have been randomly introduced along the scenario. The employed simplified human body model, implemented in house in order for it to be coupled to the 3D ray launching code, has been previously tested, exhibiting adequate performance [15, 16].

**Figure 8:**
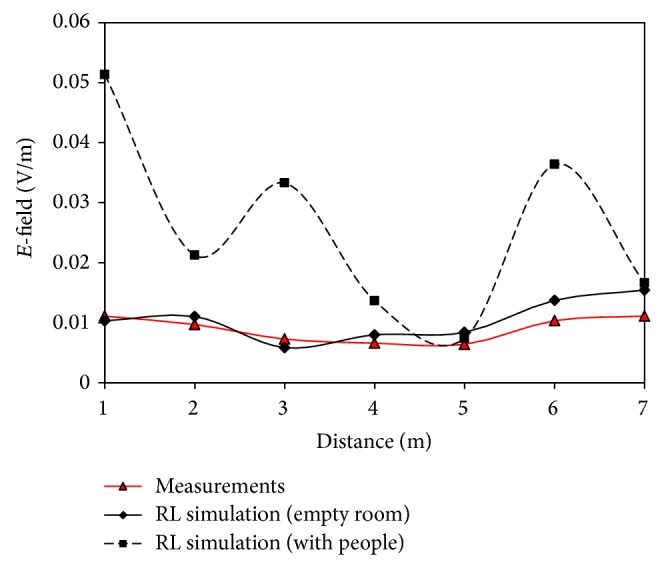
Received electric field values versus distance of measurements and ray launching simulation in empty scenario and with people considering WiFly as emitter.

In Figure 8 the comparison between measurements and simulation results is depicted. Note that the introduction of the human body inside the room changes absolutely the received power in the considered points and, in fact, the simulation results obtained in this case are far away from the real received power. As expected, the empty scenario results are similar to measures, having only a mean error of 0.002 V/m. In any case, both the measurements and simulation results comply with the recommendations proposed by ICNIRP, being the maximum allowed level of 61 V/m and 0.05 V/m the received electric field level in the worst case.

**Figure 9:**
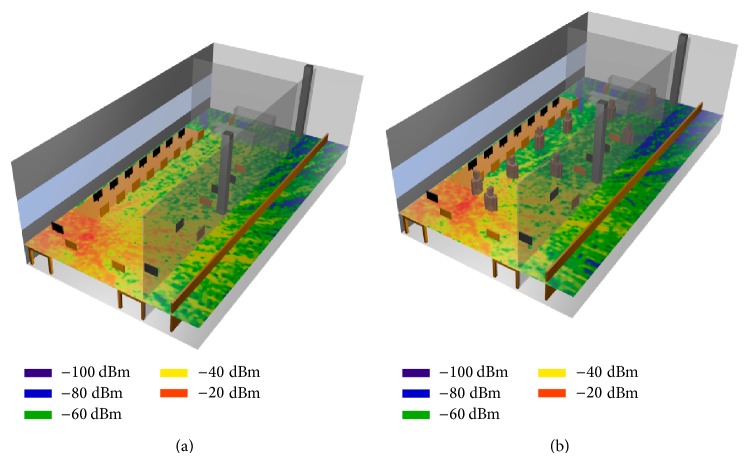
Simulated power distribution when the WiFly device is emitting without people (a) and with people (b).

The influence of the human body introduction is more visible in Figure 9 where the receiver power distribution obtained in simulation is depicted for both the empty scenario and the scenario with people inside it.

In spite of the fact that in Figure 8 the simulated points receive more power in the case where people is considered, it is visible that the received power in the global of the scenario is lower when the human body is inside it. This behavior is caused by the high absorption rate of the human body, although the ricochets produced by it could cause higher levels in some areas.

**Figure 10:**
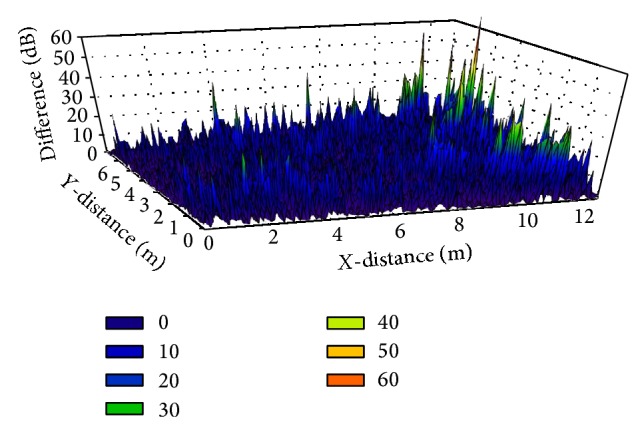
Difference between the received power distribution when the scenario is empty and when people presence is considered.

In order to stress the influence of the presence of human body models, the power difference between the two scenarios is depicted in Figure 10. As it can be seen, significant differences can be observed, due to the inclusion of the human body models, introducing high levels of absorption losses as well as other elements of interaction, such as reflection components which interact with the rest of propagating contributions within the scenario.

**Figure 11:**
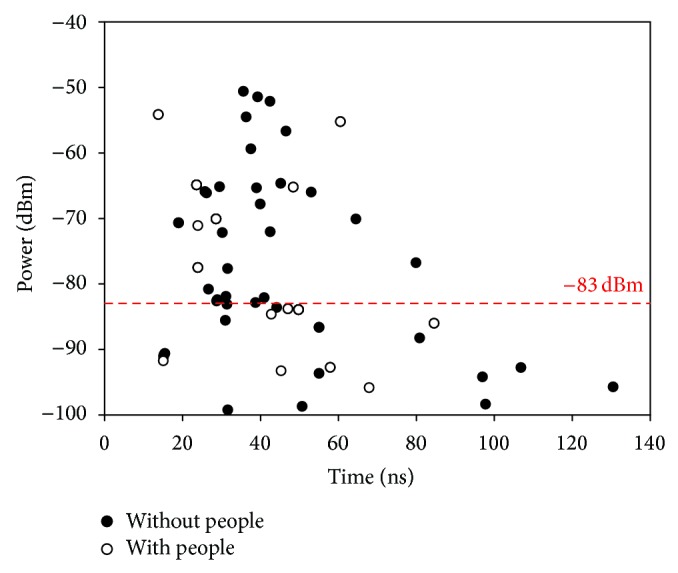
Power delay profile considering empty and full scenario with the sensitivity of the device overexposed.

In Figure 11 power delay profile estimation is depicted comparing both scenarios. The obtained results shows the number of ricochets which cross one point of the space and as expected not only is the number of bounces lower when there are human bodies inside the scenario, but also they arrive with more power when it is empty. Those results support the aforementioned hypothesis, considering that the human body has absorbed a part of the emitting power which in the other case has reached that point.

**Figure 12:**
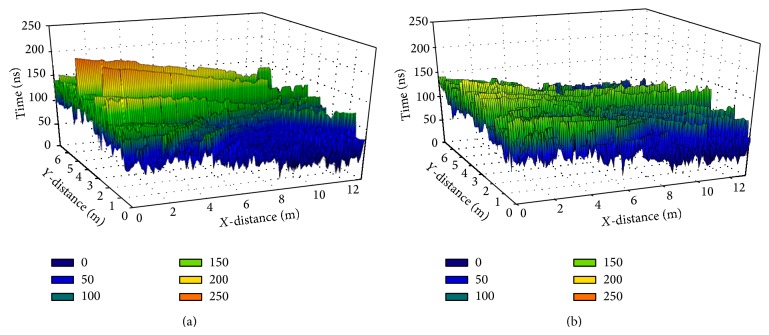
Delay Spread for the scenario without (a) and with people (b).

In order to provide more insight in the effect of the complex indoor environment the Delay Spread estimation for all points of the scenario in 0.9 m height can be shown. In both Figures 11 and 12, it is visible that the considered scenario is a very reflective room. Like it can be seen also in Figure 9, the morphological dependence of the distributed power is also demonstrable. For example, once the electromagnetic wave reach the wall situated around the principal room, the received power decreases and consequently the delay spread times either.

Finally, is important to stress again upon the influence of the people considered inside the room and the fact that most of the launched rays are absorbed by them decreasing considerably the number of rays and the delay spread time.

Once the measurements and the simulations have been compared and validated, SAR will be calculated utilizing the introduced human body models in the scenario. These values are obtained with the aid of ([Disp-formula EEq1]), an approach that has been employed in similar studies previously [17–19]. The skin properties have been considered with a value for conductivity (*σ*) of 10.18 m/s [15] and a density (*ρ*) of 1043 kg/m^3^ [20]. Consider

(1) $$\begin{array}{c} \text{SAR}=\frac{\sigma }{\rho }{\left|\vec{E}\right|}^{2}. \end{array}$$

**Figure 13:**
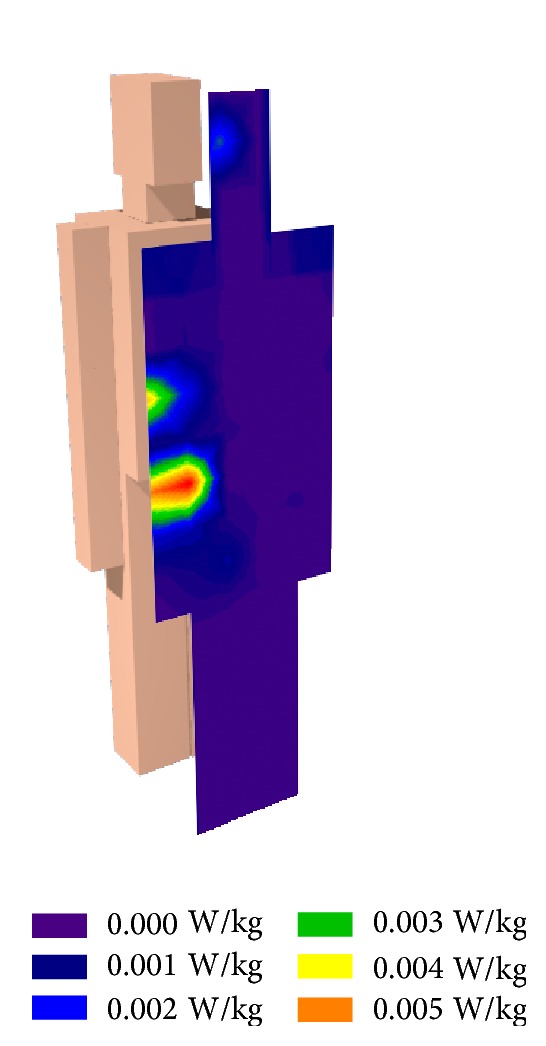
Estimation of received SAR by a human body inside the scenario.

In Figure 13, SAR values for the person who is nearest to the antenna are depicted. In this case the human body is situated in profile to the antenna and, therefore, the highest SAR values are received in the right side of the body. Nevertheless and considering the low electric field and power received in aforementioned experiments, SAR values are far away from the recommendations collected in ICNIRP guidelines [3], reaching 0.00037 W/kg mean in whole body and being 0.08 W/kg the limit value which ICNIRP recommends.

## 4. Discussion

The objective of this study is to quantify the exposure in the proximity of the device due to the increasing use of Wi-Fi wireless devices that operate in the band of 2.4 GHz and to analyze the compatibility between equipment and networks in different environments.

For the near field setup, the lower sensitivity limit of the E-field probe (2 V/m) has not been reached. The value of the E-field is 27.1 V/m; this involves that the more restrictive threshold of 3 V/m, established in the International Electrotechnical Commission Standard of Electromedical Devices [8], is exceeded. It is important to consider that the device under test was transmitting information continuously while the measurement campaign have been carried out, which means a duty factor of 100%. The duty factor is referred to as the relation between the time interval of effective transmission and the total duration of the transmission. Usually, Wi-Fi devices do not transmit information continuously, depending on traffic demands, adaptive modulation and coding schemes and additional quality of service constraints. It has been documented that exposure levels of the EM field depend on the data rate at which the information is being transmitted [21]. Regarding the influence of wireless local area networks (WLAN), no electromagnetic interference caused by the WLAN technology was documented by using in vitro testing of pacemakers and implantable cardioverter defibrillators (ICD) [22]. In order to avoid medical device malfunction, it is recommended to maintain a distance from the transmitting device greater than 1 m.

It is worth noting that all the field strengths recorded in this study are well below the corresponding ICNIRP reference level of 61 V/m defined for the general public at the working frequency (2.4 GHz) [3].

The near field data is complemented with the analysis provided of interaction of devices and users in an indoor scenario in which complex topomorphological considerations can be taken into account. Due to the interaction with the elements within the environment, strong multipath components appear which modify the emission levels from the WiFly device. In the case of analyzing emissions not colocated in the human body, estimated field levels are in compliance with exposure guidelines, given by strong attenuation of the propagated signal mainly due to multipath components. Moreover, the location of potential transceivers as well as the distribution of the indoor environment plays a key role in the observable power distributions, as well as the inclusion of adequate human body models.

## 5. Conclusion

Wireless transceivers will play a fundamental role in the adoption of context aware environment, with application in multiple scenarios such as building automation, ambient assisted living, e-health, and s-health environments, among others. In this sense, the characterization of a Wi-Fi transceiver has been performed both in near field as well as in a full indoor complex scenario, emulating the real behavior of this device. The results find application both in electromagnetic exposure as well as in interference assessment.

Measurements have been realized in an anechoic chamber with the measurement system DAISY5PRO that is provided with the possibility of measure the E-field in predefined and programmed positions. Under usual operating conditions the levels of E-field caused by the tested social alarm device do not exceed the limits of personal exposure according to ICNIRP 1998 [3]. In the cases of very small distances from the tested device, the highest level of E-field strength exceeds the more restrictive threshold of 3 V/m that is established in the International Electrotechnical Commission Standard of Electromedical Devices [8]. The measurement conditions are characterized by a duty factor of the transmission of 100%, while in real conditions the duty factor is considerable lower. In practice the duty factor does not usually exceed 65%, hence providing estimations for the worst case.

The analysis has also been extended to a larger indoor scenario, with the aid of an in-house implemented deterministic 3D ray launching code, in which scenario elements as well as human body models have been included. By following this approach, estimations can be obtained in large complex scenarios maintaining an adequate compromise between accuracy and computational complexity. The results show that emission levels are in principle in compliance with emission level guidelines, although capable of introducing nondesired components which could act as interferers to other systems. Therefore, taking into account the role of wireless transceivers in the advent of context aware scenario, where massive deployments are expected, electromagnetic emission analysis by means of deterministic modelling can aid in providing overall interference and exposure assessment. The proposed technique, tested on a Wi-Fi device, can be readily extended to include multiple systems, such as IEEE 802.15 devices or 3G/4G mobile devices.

## Figures and Tables

**Table 1:** Ray launching simulation parameters.

Parameter	Value
Frequency of operation	ISM 2.4 GHz
Radiation pattern	Monopole
Transmitted power	10 dBm
Resolution (cuboids size)	10 cm × 10 cm × 10 cm
Maximum reflections permitted	5
Vertical and horizontal launched rays resolution	1°

## References

[1] Solanas A., Patsakis C., Conti M. (2013). Smart health: a context-aware health paradigm within smart cities.

[2] Soomro A., Cavalcanti D. (2007). Opportunities and challenges in using WPAN and WLAN technologies in medical environments. *IEEE Communications Magazine*.

[3] International Commission on Non-Ionizing Radiation Protection (1998). Guidelines for limiting exposure to protection time-varying electric, magnetic, and electromagnetic fields (up to 300 GHz). *Health Physics*.

[4] Barbiroli M., Carciofi C., Guiducci D. (2011). Assessment of population and occupational exposure to Wi-Fi systems: measurements and simulations. *IEEE Transactions on Electromagnetic Compatibility*.

[5] De Miguel-Bilbao S., Martín M. A., Del Pozo A. (2013). Analysis of exposure to electromagnetic fields in a healthcare environment: simulation and experimental study. *Health Physics*.

[6] Directive 2013/35/EU of the European Parliament and of the Council On the minimum health and safety requirements regarding the exposure of workers to the risks arising from physical agents (electromagnetic fields) (20th individual Directive within the meaning of Article 16(1) of Directive 89/391/EEC) and repealing Directive 2004/40/EC.

[7] de Miguel-Bilbao S., Roldán J., García J., Ramos V., Fernández J., Suárez O. J. Assessment of exposure from Wi-Fi devices.

[8] International Electrothecnical Commission (IEC)

[9] Hata M. (1980). Empirical formula for propagation loss in land mobile radio services. *IEEE Transactions on Vehicular Technology*.

[10] Ikegami F., Yoshida S., Takeuchi T., Umehira M. (1984). Propagation factors controlling mean field strength on urban streets. *IEEE Transactions on Antennas and Propagation*.

[11] Dimitriou A. G., Sergiadis G. D. (2006). Architectural features and urban propagation. *IEEE Transactions on Antennas and Propagation*.

[12] Azpilicueta L., Falcone F., Astráin J. J. (2012). Measurement and modeling of a UHF-RFID system in a metallic closed vehicle. *Microwave and Optical Technology Letters*.

[13] Nazabal J. A., Iturri P. L., Azpilicueta L., Falcone F., Fernández-Valdivielso C. (2012). Performance analysis of IEEE 802.15.4 compliant wireless devices for heterogeneous indoor home automation environments. *International Journal of Antennas and Propagation*.

[14] Iturri P. L., Nazábal J. A., Azpilicueta L. (2012). Impact of high power interference sources in planning and deployment of Wireless Sensor Networks and devices in the 2.4 GHz frequency band in heterogeneous environments. *Sensors*.

[15] Aguirre E., Arpón J., Azpilicueta L., de Migue S., Ramos V., Falcone F. (2012). Evaluation of electromagnetic dosimetry of wireless systems in complex indoor scenarios with human body interaction. *Progress in Electromagnetics Research B*.

[16] Led S., Azpilicueta L., Aguirre E., de Espronceda M. M., Serrano L., Falcone F. (2013). Analysis and description of HOLTIN service provision for AECG monitoring in complex indoor environments. *Sensors*.

[17] Sánchez-Hernández D. A. (2009). *High Frequency Electromagnetic Dosimetry*.

[18] Loader B., Gregory A., Bownds D., Seifert F. Evaluation of an optical electric field sensor for measurement of specific absorption rate (SAR) during magnetic resonance imaging.

[19] Kininami K., Iyama T., Onishi T., Uebayashi S. (2008). Evaluation of an optical electric field sensor for measurement of specific absorption rate (SAR) during magnetic resonance imaging. *IEEE Transactions on Electromagnetic Compatibility*.

[20] Krzywicki H. J., Chinn K. S. (1967). Human body density and fat of an adult male population as measured by water displacement. *The American Journal of Clinical Nutrition*.

[21] Peyman A., Khalid M., Calderon C. (2011). Assessment of exposure to electromagnetic fields from wireless computer networks (wi-fi) in schools; results of laboratory measurements.. *Health physics*.

[22] Tri J. L., Trusty J. M., Hayes D. L. (2004). Potential for personal digital assistant interference with implantable cardiac devices. *Mayo Clinic Proceedings*.

